# Enterprise size and risk of hospital treated injuries among manual construction workers in Denmark: a study protocol

**DOI:** 10.1186/1745-6673-6-11

**Published:** 2011-04-21

**Authors:** Betina H Pedersen, Harald Hannerz, Ulla Christensen, Finn Tüchsen

**Affiliations:** 1 National Research Centre for the Working Environment, Copenhagen, Denmark; 2 Department of Public Health, Section for Social Medicine, University of Copenhagen, Denmark

## Abstract

**Background:**

In most countries throughout the world the construction industry continues to account for a disturbingly high proportion of fatal and nonfatal injuries. Research has shown that large enterprises seem to be most actively working for a safe working environment when compared to small and medium-sized enterprises. Also, statistics from Canada, Italy and South Korea suggest that the risk of injury among construction workers decreases with enterprise size, that is the smaller the enterprise the greater the risk of injury. This trend, however, is neither confirmed by the official statistics from Eurostat valid for EU-15 + Norway nor by a separate Danish study - although these findings might have missed a trend due to severe underreporting. In addition, none of the above mentioned studies controlled for the occupational distribution within the enterprises. A part of the declining injury rates observed in Canada, Italy and South Korea therefore might be explained by an increasing proportion of white-collar employees in large enterprises.

**Objective:**

To investigate the relation between enterprise size and injury rates in the Danish construction industry.

**Methods/Design:**

All male construction workers in Denmark aged 20-59 years will be followed yearly through national registers from 1999 to 2006 for first hospital treated injury (ICD-10: S00-T98) and linked to data about employment status, occupation and enterprise size. Enterprise size-classes are based on the Danish business pattern where micro (less than 5 employees), small (5-9 employees) and medium-sized (10-19 employees) enterprises will be compared to large enterprises (at least 20 employees). The analyses will be controlled for age (five-year age groups), calendar year (as categorical variable) and occupation. A multi-level Poisson regression will be used where the enterprises will be treated as the subjects while observations within the enterprises will be treated as correlated repeated measurements.

**Discussion:**

This follow-up study uses register data that include all people in the target population. Sampling bias and response bias are thereby eliminated. A disadvantage of the study is that only injuries requiring hospital treatment are covered.

## Background

Injuries related to construction work remain a serious problem worldwide. Although many prevention efforts and intervention programs have been undertaken [1,2], it is a known fact that construction workers continue to carry a particularly high risk of sustaining fatal and nonfatal injuries. The International Labour Organization (ILO) estimates that more than 100,000 construction workers around the world die every year - that is one person every five minutes [3]. In the European Union (EU-15 + Norway), workers employed in the construction sector from 1995 to 2005 showed the second highest incidence rate of fatal injuries at work and the highest incidence rate of nonfatal injuries at work [4]. Yet an overall trend of improvement is worth noting, as the total number of injuries in construction dropped 16% over the ten-year period. Still, the serious human and socio-economic consequences of approximately 1,000 European construction workers dying every year from work-related injuries and the 30,000 getting so severely disabled that they can no longer work, call for action [4(year 2005 figures)].

In EU's strategic plan for reducing the number of work-related injuries with 25% from 2007 to 2012, the construction sector is intelligibly stressed as particularly dangerous. Moreover, small and medium-sized enterprises (SMEs) - which according to EU are defined as enterprises with 10-49 and 50-249 employees, respectively - are considered to be especially vulnerable workplaces in terms of guaranteeing a healthy and safe working environment [5]. Several studies have underlined an elevated injury risk in SMEs [6-9]. One obvious reason for SMEs to be considered such risky and harmful workplaces is that they typically have fewer financial, human and technological resources available for organization and management of safety and health precautions. Economic survival and economic competition concerns quite often might override basic health and safety concerns. Another reason is that SMEs often seem to be lacking the ability to perform proactive or high-quality risk management [10-14]. In addition, the owner's reluctance towards state regulation of employees' health and safety issues seems to be decisive [15]. So in general large enterprises seem to most actively make an effort in ensuring a safe and sound working environment when compared to small and medium-sized enterprises.

The business pattern in the European Union is composed of 99% SMEs of which 92% are micro enterprises with less than 10 employees [16]. The investigation of construction SMEs, and especially micro enterprises, and their challenges to perform safety and health improvements thus continues to be of utmost importance for public health. Recent statistics from the European Commission from year 2005 show a higher injury incidence rate of around 6,500 per 100,000 construction workers in SMEs compared to an injury incidence rate of 4,700 per 100,000 construction workers in large enterprises. For fatalities in construction, the incidence rate is around 9 per 100,000 in SMEs while 5.5 for large enterprises [4].

The information obtained through comparisons of large enterprises (more than 250 employees) and SMEs (1 to 249 employees) is, however, not very useful in a Danish context. In Denmark the vast majority of enterprises, 92%, employ fewer than 10 workers, but only 2% employ more than 50 workers [17]. When looking at the Eurostat injury data for the relation between each enterprise size and not only SMEs against large enterprises in the construction industry, data do not indicate that injury rates increase with enterprise size among enterprises with less than 250 employees [4]. In fact, the reported injury rates among enterprises with 1-9 employees were lower than enterprises with 10-49 employees in each of the studied calendar years (1996-2005). EU's injury data, however, are subject to underreporting because data, in the case of Denmark, are based on injury reports to the National Labour Inspectorate exclusively lodged by employers.

Kines & Mikkelsen (2003) attempted to investigate rates of elevation fall injuries as a function of enterprise size in the Danish construction industry in the years 1993-1999 [18]. They used the following enterprise categorization: 0; 1-4; 5-9; 10-19; 20-49; 50-99; and > = 100 employees, yet their results were inconclusive; no noticeable trend was found. But as was noted by the authors, the investigation was possibly biased due to an underreporting of approximately 50% of the injuries. Moreover, enterprise size was not given in 13% of the reported injuries. Another problem with the study was that there was no control for the occupational distribution within the enterprises. According to Danish national data, injury rates among blue collar workers are on average twice as high as they are among white collar workers [19]. Also, it has been shown that injury rates differ between occupational categories among blue collar construction workers [20].

In contrast, three studies do indicate a trend of decreasing injury rates with enterprise size in the construction industry valid for the following settings: Ontario, Canada in the years 1988 to 1993 [21], South Korea in the years 1991 to 1994 [22], and Italy in the years 1995 to 2000 [8]. None of these three studies controlled for occupational distribution within the enterprises; hence, at least part of the decline in injury rates might be due to an increasing proportion of white-collar employees in the larger enterprises.

The primary aim of the present study is to investigate the relation between enterprise size and injury rates in the Danish construction industry, on a data set that is free from reporting bias, while controlling for the occupational distribution within the enterprises. A conditional aim will be to examine if there is an association between a change in Danish legislation and injury rates among construction workers in enterprises with 5-9 employees. The change, which was implemented from July 1, 2002, cancelled the requirement to have a safety organisation for enterprises with 5 - 9 employees and may have resulted in a higher risk of injury in these enterprises.

While performing the primary analysis, we will take the opportunity to estimate relative injury rates in the occupational groups that are included in the analysis. A special attention will be given to the rates among bricklayers, carpenters and plumbers whose work environment we plan to scrutinise in a subsequent project funded by the same grant as the present work.

## Methods/Design

The study is designed as an observational analytical population study. The population is dynamic (open for both entry and departure) and consists of all male construction workers in Denmark aged 20-59 years during the study period January 1, 1999 to December 31, 2006. The start of the study period coincides with the launching of the registration of all local workplace units in the Central Business Register in Denmark - a national register which contains primary data on all public and private businesses. The end of the study period refers to the year of the latest statistical returns on workplace size linked to local workplace unit from Statistics Denmark.

The subjects are followed one year at a time for first hospital treated injury during the year. The injuries are diagnosed on the basis of ICD-10 classification numbers S00-T98: "Injury, poisoning and certain other consequences of external causes" [23]. Included in the study are principal diagnoses as concluded either by discharge from the hospital, or by transfer to another hospital division.

### Data sources and classifications

The Danish Occupational Hospitalisation Register (OHR) is used to identify injured individuals. Included in the OHR are all persons who have been a legal/registered inhabitant of Denmark, aged 20 or more, at one time or another since 1980. OHR consists of a record-linkage between three national registers: 1) the central person register, 2) the national hospital patient register, and 3) the employment classification module.

The central person register contains information on gender, addresses, and dates of birth, death and migrations for everyone registered as living in Denmark sometime from 1968 to present.

The national hospital patient register contains data from all public hospitals in Denmark. Patient diagnoses have been coded according to the international classification of diseases version ten (ICD-10) since 1994. Since 1995, the register has covered all inpatients, outpatients, and emergency ward visits [24]. Relevant for the present study is that, in the follow-up period, no private emergency wards existed in Denmark, and that less than 1% of all planned surgery on in- and outpatients took place in private hospitals [25].

The employment classification module contains annually registered information on a person's industry, occupation, and employment status from 1975 onwards [24]. For the time-period spanned by the present study, the industries were initially coded according to the 1993 and then to the 2003 version of the Danish Industrial Classification of All Economic Activities [26,27]. These classification systems are national versions of the European Industrial Classification of All Economic Activities (NACE rev. 1). NACE rev.1 divides industries hierarchically into 17 level-1 sections identified by alphabetical letters A to U; 60 level-2 divisions identified by two-digit numerical codes (01 to 99); 222 level-3 groups identified by three-digit numerical codes (01.1 to 99.0), and 503 level-4 classes identified by four-digit numerical codes (01.11 to 99.00). In the present study only levels 1 and 2 are used; at level 1, the letter "F" refers to the construction industry and its level 2 number is "45".

The occupations in the employment classification module were coded according to DISCO-88 [28], which is a national version of the international standard classification of occupations (ISCO-88) [29]. DISCO-88 divides the occupations hierarchically into 10 major groups; 27 sub-major groups; 111 minor groups, and 372 unit groups. Included in the present study are the major groups related to manual construction work: group 7 "Craft and related trades workers"; group 8 "Plant and machinery operators and assemblers", and group 9 "Elementary occupations".

OHR-data of each injured individual will be linked to the latest national statistical returns of workplace size and local workplace unit. The statistical returns are assessed by Statistics Denmark every year in week 48, i.e. the last week in November, and imply that the employment data about each injured individual in the population stem from the year before the hospital treatment of the injury. Data about workplace size identifies the number of employees in addition to the owner of the workplace. Data about workplace unit identifies the local workplace unit where the injured individual was mainly carrying out his job. The local workplace unit can be the exact same as the mother enterprise unit, or it can be a unit belonging to the mother enterprise, but with a different geographical location and therefore with a different unit number. If a person worked in more than one place, which is often the case for construction workers, the local workplace unit is taken to be the workplace from where instructions emanate, or from where the work is organised.

Records are linked by means of a unique personal identification number and are kept at Statistics Denmark. Researchers are authorized to use data with encrypted personal identification numbers, and it is secured so that no analyses identifying any person or enterprise can be transferred outside Statistics Denmark.

### Study population

Inclusion criteria for the study population are:

• main employment in the construction industry (NACE code = '45');

• employment status as employee or self-employed, that is with the highest income as such during the year;

• job function as manual worker (DISCO-88 code = 7, 8 or 9);

• age from 20 to 59 years - the former time limit due to available OHR-data from the age of 20; the latter time limit due to the possibility of job release scheme from the age of 60;

• male worker - the women will be left out of the study since they constitute less than four percent of the blue-collar workers of the construction industry.

A person enters the population as soon as all of the above criteria are fulfilled, and departs whenever they are no longer met.

### Statistical analyses

A person will become a case once receiving a principal diagnosis in the ICD-10 interval S00-T98 ("injury, poisoning and certain other consequences of external causes") according to the OHR. For any given calendar year, a person will be censored at the time he becomes a case, emigrates or dies. Time-dependent dummy variables are used to categorise the manual workers into micro enterprises (fewer than 5 employees), small enterprises (5-9 employees), medium-sized enterprises (10-19 employees), and large enterprises (20 or more employees). A person's work category during a certain calendar year is determined by his enterprise association according to the population census performed in the end of November the preceding year.

The null hypothesis stating that "the injury rates among workers are independent of enterprise size" will be tested. If this first null hypothesis is rejected meaning that the observed injury rates most likely depend on enterprise size, a second null hypothesis will be tested. This second null hypothesis will test if "the relative rate of injury among workers in enterprises with 5-9 employees compared with other workers is independent of time period (January 1, 1999 - June 30, 2002 versus July 1, 2002 - December 31, 2005)". By this, it shall be tested if it can be assumed that the legislative change that took place in Denmark on 1 July, 2002, which cancelled the requirement of having a safety organisation in enterprises with 5 - 9 employees, did not have any effect on the injury rates among the workers in enterprises with 5-9 employees.

To deal with intra-enterprise correlations, a multi-level Poisson regression will be used to model the outcome, where the enterprises will be treated as the subjects while observations within the enterprises will be treated as correlated repeated measurements.

The analyses will be controlled for age (five-year age groups), calendar year (as categorical variable) and occupation. Occupation unit groups are: Bricklayers and stonemasons (DISCO-88 = 7122); Carpenters and joiners (DISCO-88 = 7124); Plumbers and pipe fitters (DISCO-88 = 7136); Electricians (DISCO-88 = 7137); Painters and wall-paper workers (DISCO-88 = 7141); Unskilled manual workers in construction workers (DISCO-88 = 9313).

The analyses will be performed by use of the GENMOD procedure in SAS version 9.1. Only main effects will be considered. The empiric standard error estimates will be used and an exchangeable correlation structure is assumed. The significance level will be set to 0.05. Table 1 shows the rate ratios and 95% confidence intervals that will be calculated.

**Table 1:** Manual construction workers' relative risk rates of injury, by enterprise size and type of profession

	Injury Rate Ratio	Confidence Interval (95% CI)
**Enterprise size in construction (P = xxxx)**		

**Micro vs. large**		

**Small vs. large**		

**Medium-sized vs. large**		

**Type of construction profession (P = xxxx)**		

**Bricklayers and stonemasons vs. other manual construction workers**		

**Carpenters and joiners vs. other manual construction workers**		

**Plumbers and pipe fitters vs. other manual construction workers**		

**Electricians vs. other manual construction workers**		

**Painters and wall-paper workers vs. other manual construction workers**		

**Unskilled construction workers vs. other manual construction workers**		

## Discussion

Since this is not a randomized study, we cannot rule out that selection into the enterprises may influence the estimates in the sense that cautious people, for example, might prefer employment in large enterprises where safety more often seems to be a priority and different regulatory requirements for safety leads to fewer risk situations. Whereas more reckless people who care less about the potential dangers, or may thrive better with their own risk perception as they themselves decide when to take precautions relative to safety, might prefer employment in small enterprises where fewer legal requirements for safe work must be respected. The effect of enterprise size would then be intensified by the effect of personality type and would bias the estimates away from unity. Conversely, a selection bias towards unity would be the case if, for example, an owner of a micro enterprise focuses on avoiding human and economic losses caused by work-related injuries and therefore is carefully seeking to recruit diligent workers. Moreover, workers in a micro enterprise are probably in closer contact to the management (or owner) compared with those working in a large enterprise and such a close proximity would make it easier for the management to detect risky behaviour in the workplace and dismiss it before injuries occur. We believe, however, that our study group is far more homogeneous than those in most other occupational risk studies and this may counteract potential selection bias. All of the included workers are manual workers belonging to the same industry and most of them are belonging to the same occupational class (skilled workers). The exception is the occupational group 'unskilled construction workers', but this will be controlled for in the analysis.

The Occupational Hospitalisation Register is free of reporting bias. All hospital contacts are registered, and there are virtually no missing principal diagnoses. This can be contrasted with two alternative national data sources held by the Danish Working Environment Authority and the National Board of Industrial Injuries, respectively, to whom merely 45% of all work-related injuries are reported [30]. Another advantage of the study is that we have identification numbers on the people as well as the enterprises, which enables us to take intra-enterprise correlations into account. Since the local workplace unit number is based on each geographically bounded production unit, this study provides more precise information about workplace size than the mother company number which merely sums up the number of employed for all subordinated workplaces. The study is further strengthened by its sample size, which will afford sufficient statistical power to the various hypothesis tests.

A disadvantage of the Occupational Hospitalisation Register is that it leaves out injuries that do not need hospital treatment. Another disadvantage is that we have annual but not daily information about occupation and enterprise. Enterprise association at the time of the injury might be different from the enterprise association in the end of November the preceding calendar year and the occupational category might change during the course of the year. We believe that most of the occupational categories are quite stable since we are dealing with skilled workers. It would, for example, be unlikely that a trained carpenter would shift into a brick layer trainee in the course of a calendar year. The construction industry is, however, known for its high turnover rate [31]. Within a calendar year, a worker in a small enterprise might, for example, move to a large enterprise, where he is injured. According to our model, such an injury would erroneously be classified as having happened in a small enterprise. The size of an enterprise may also change during a calendar year. All such changes would bias our estimates toward unity; from this perspective, the estimates should be regarded as conservative. As such, we hope that our study will contribute to a better assessment of relative injury rates in small and medium-sized enterprises.

## List of abbreviations used

SMEs: Small and medium-sized enterprises: Firstly, the term "SME" contains micro, small, and medium-sized enterprises. Secondly, in this study the distinction of the enterprise size-classes is based on the Danish business pattern: Micro enterprises include the self-employed and enterprises with fewer than 5 employees, small enterprises have 5 to 9 employees; medium-sized enterprises have 10 to 19 employees; and large enterprises employ at least 20 persons. In a European context, SMEs are distinguished as: Micro enterprises with fewer than 10 employees, small enterprises with 10 to 49 employees, and medium-sized enterprises with 50 to 249 employees [16]; EU: The European Union. At present (2010), EU has 27 member states. By 2004, Cyprus, Czech Republic, Estonia, Hungary, Latvia, Lithuania, Malta, Poland, Slovakia, and Slovenia joined the EU. By 2007, Bulgaria, Romania joined the EU; EU-15: Austria, Belgium, Denmark, Finland, France, Germany, Greece, Ireland, Italy, Luxembourg, the Netherlands, Portugal, Spain, Sweden and the United Kingdom; ICD-10: International Classification of Diseases version number 10. ICD-10 was endorsed by the Forty-third World Health Assembly in May 1990 and came into use in WHO Member States as from 1994; OHR: The Danish Occupational Hospitalisation Register; DISCO-88: National version of the international standard classification of occupations (ISCO-88); NACE rev. 1: Statistical classification of economic activities in the European community from 1 January 1993. The word NACE is a French acronym for "Nomenclature generale des Activites economiques dans les Communautes Europeennes", the first classification from 1970 covering the whole range of economic activity.

## Competing interests

The authors declare that they have no competing interests.

## Ethics approval

The study will comply with The Act on Processing of Personal Data (Act No. 429 of 31 May 2000), which implements the European Union Directive 95/46/EC on the protection of individuals. The data usage is approved by the Danish Data Protection Agency, journal number: 2001-54-0180. According to Danish law, questionnaire and register based studies do not need approval by ethical and scientific committees, nor informed consent.

## Authors' contributions

BHP and HH designed the study and prepared the first draft of the manuscript. All authors contributed in a critical revision of the manuscript. All authors have given their final approval of the version submitted for publication.
